# Supplementary material for: Combining gut microbiota modulation and chemotherapy by capecitabine-loaded prebiotic nanoparticle improves colorectal cancer therapy
Source: Nat Commun. 2023 Aug 7;14:4746. doi: 10.1038/s41467-023-40439-y (PMC10406894; doi:10.1038/s41467-023-40439-y)
Supplement: Supplementary file 1 — Supplementary Information [file 41467_2023_40439_MOESM1_ESM.pdf]

Supplementary Information for:

Combining gut microbiota modulation and chemotherapy by capecitabine-loaded prebiotic nanoparticle improves colorectal cancer therapy

Tianqun Lang[1,2,#], Runqi Zhu[1,3,#], Xiao Zhu[1,3], Wenlu Yan[1,3], Yu Li[4], Yihui Zhai[1,3], Ting Wu[5], Xin Huang[1,3], Qi Yin[1,2,3]★, Yaping Li[1,2,3,4,6]★

[1] State Key Laboratory of Drug Research & Center of Pharmaceutics, Shanghai Institute of Materia Medica, Chinese Academy of Sciences, Shanghai 201203, China

[2] Yantai Key Laboratory of Nanomedicine & Advanced Preparations, Yantai Institute of Materia Medica, Shandong 264000, China

[3] University of Chinese Academy of Sciences, Beijing 100049, China

[4] School of Chinese Materia Medica, Nanjing University of Chinese Medicine, Nanjing 210023, China

[5] Department of Pharmaceutics, School of Pharmacy, Nanjing Medical University, Nanjing 211116, China

[6] Shandong Laboratory of Yantai Drug Discovery, Bohai rim Advanced Research Institute for Drug Discovery, Yantai 264117, China

[#] These authors contributed equally: Tianqun Lang, Runqi Zhu

[★] Corresponding authors: Prof. Qi Yin (qyin@simm.ac.cn); Prof. Yaping Li (ypli@simm.ac.cn); Tel/Fax: +86-21-2023-1979.

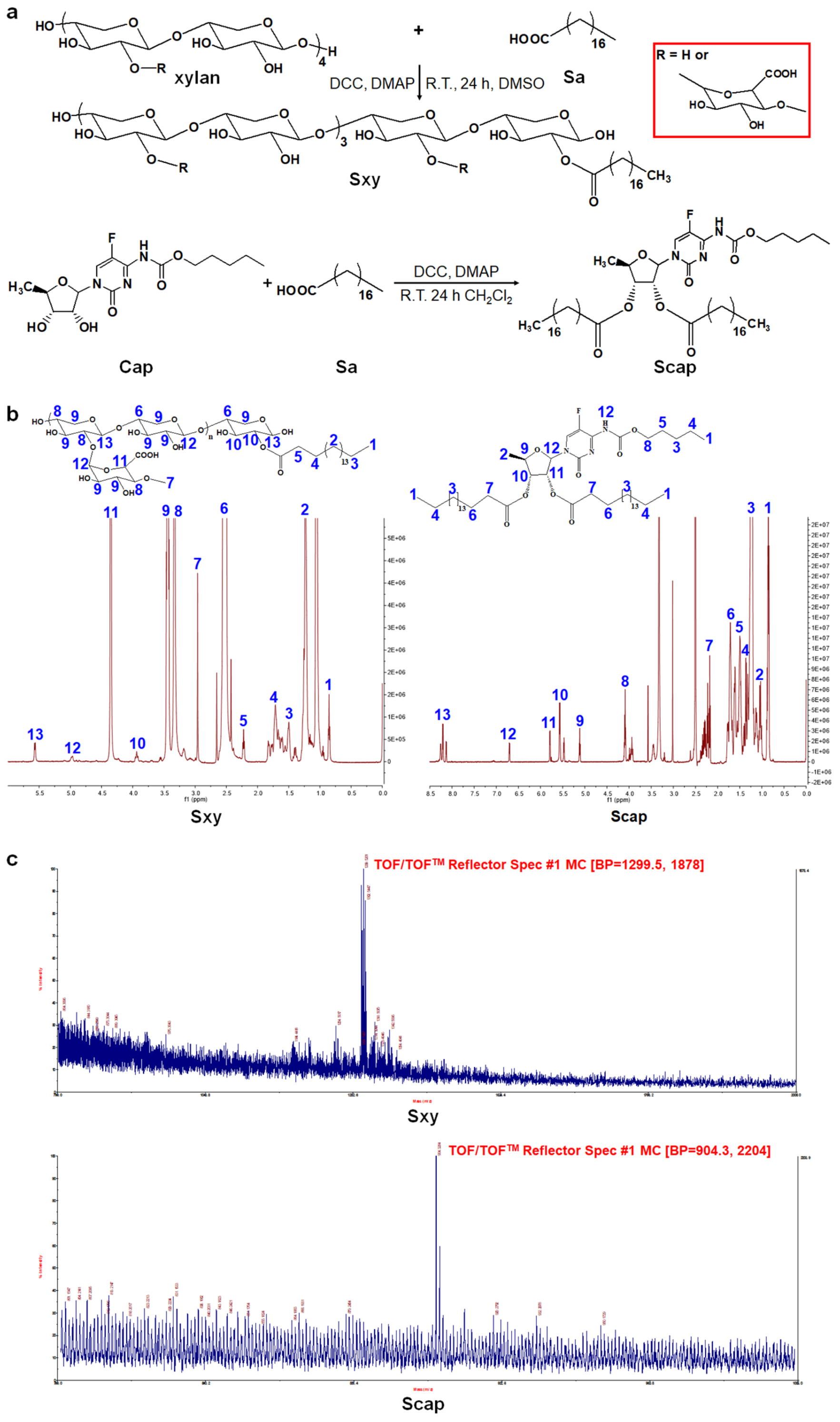

Supplementary Figure 1. Synthesis and structure confirmation of Sxy and Scap. **a,** The diagram of the synthesis of Sxy and Scap. **b,** The ^{1}H NMR spectra of Sxy and Scap. **c,** The MALDI-TOF-MS spectra of Sxy and Scap.

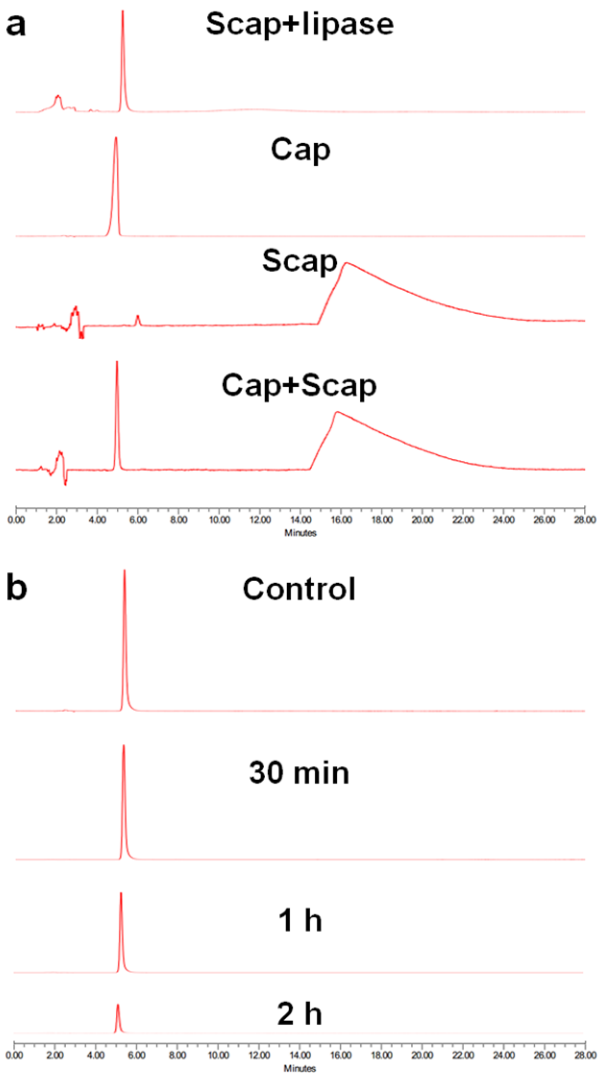

Supplementary Figure 2. Hydrolysis of Scap. **a,** The spectra of Scap after or before incubation with lipase, Cap, and Cap+Scap mixture detected by HPLC. **b,** The spectra of liver extracts taken from the mice at 30 min, 1 h, and 2 h post oral administration with Scap. The Cap solution mixed with the liver extract from the untreated mice was detected as control.

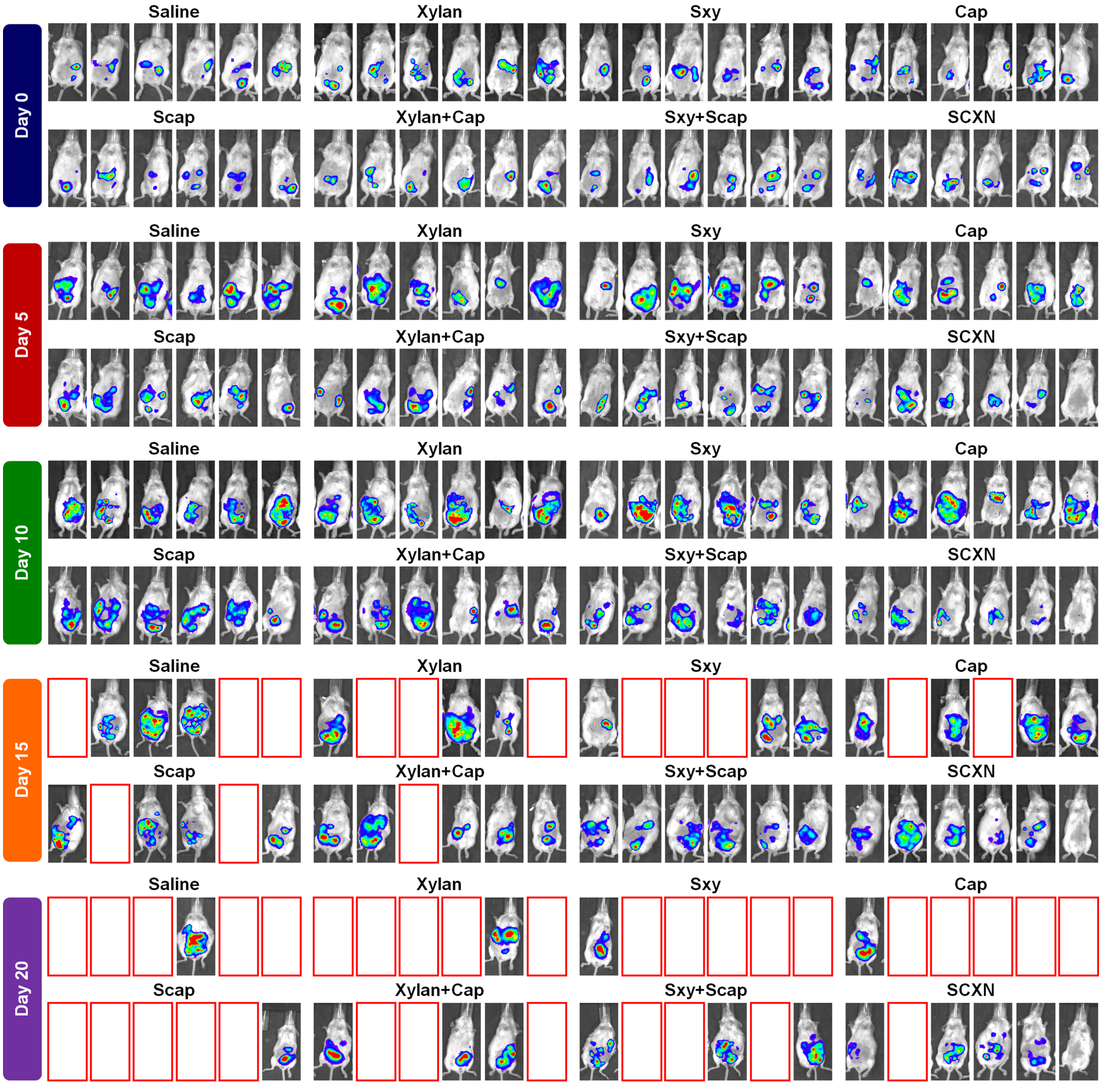

Supplementary Figure 3. In vivo bioluminescence imaging of CT26-luc tumor-bearing mice receiving mutli-dose of different formulations. n = 6 mice.

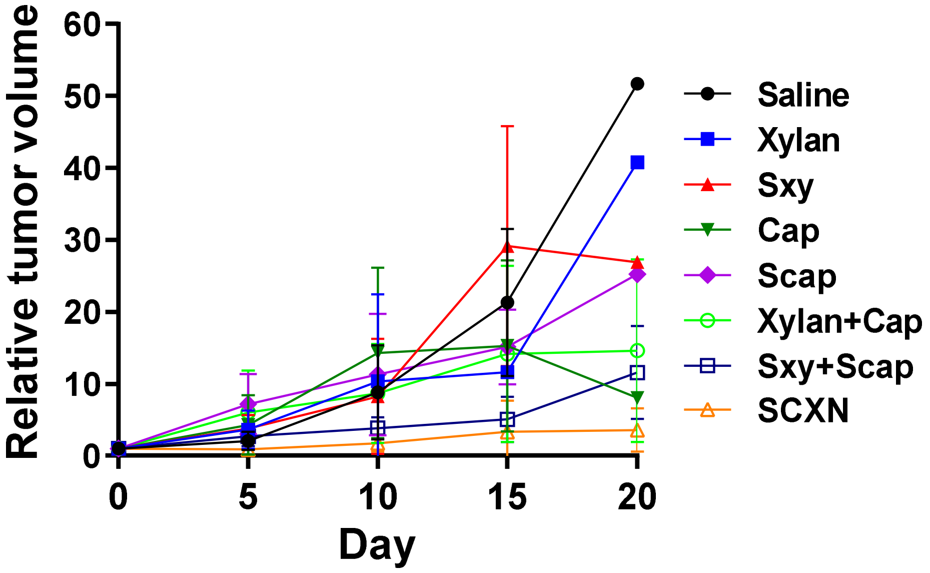

Supplementary Figure 4. The variation of relative tumor volumes in the CT26-luc tumor-bearing mice calculated according to the photons amount detected by IVIS during the therapy period. Data are shown as mean ± SD. n equals to the number of surviving mice in each group, of which the maximum value is 6. On day 15, n = 3 for Saline, Xylan and Sxy group, n = 4 for Cap and Scap group, n = 5 for Xylan+Cap group; On day 20, n=1 for Saline, Xylan, Sxy, Cap and Scap group, n = 3 for Xylan+Cap and Sxy+Scap group, n=5 for SCXN group. For all of other points, n = 6. Source data are provided as a Source Data file.

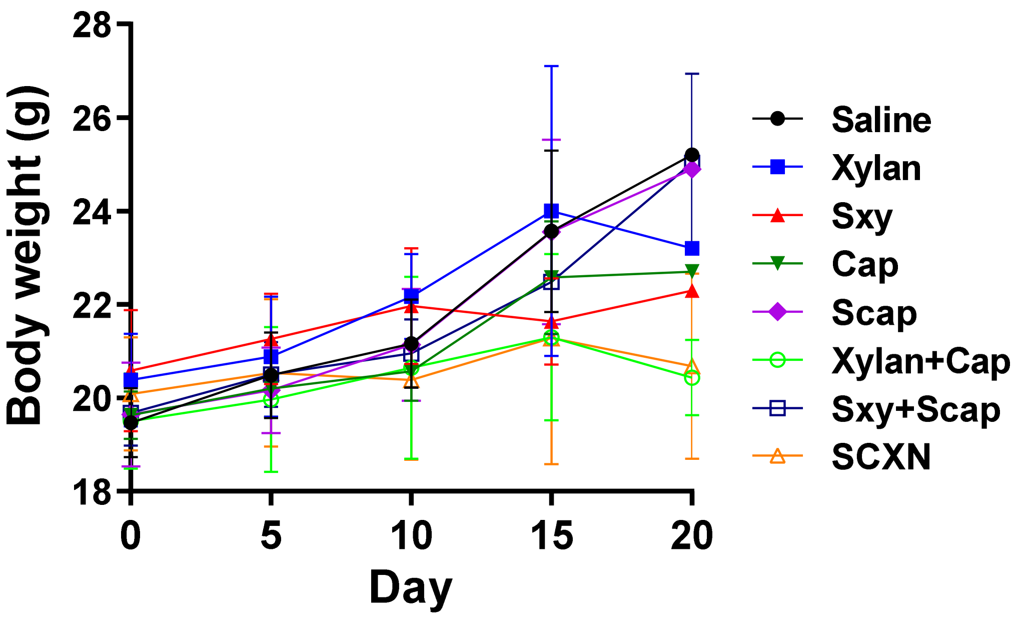

Supplementary Figure 5. The variation of average body weights of the CT26-luc tumor-bearing mice during the therapy period. Data are shown as mean ± SD. n equals to the number of surviving mice in each group, of which the maximum value is 6. On day 15, n = 3 for Saline, Xylan and Sxy group, n = 4 for Cap and Scap group, n = 5 for Xylan+Cap group; On day 20, n=1 for Saline, Xylan, Sxy, Cap and Scap group, n = 3 for Xylan+Cap and Sxy+Scap group, n=5 for SCXN group. For all of other points, n = 6. Source data are provided as a Source Data file.

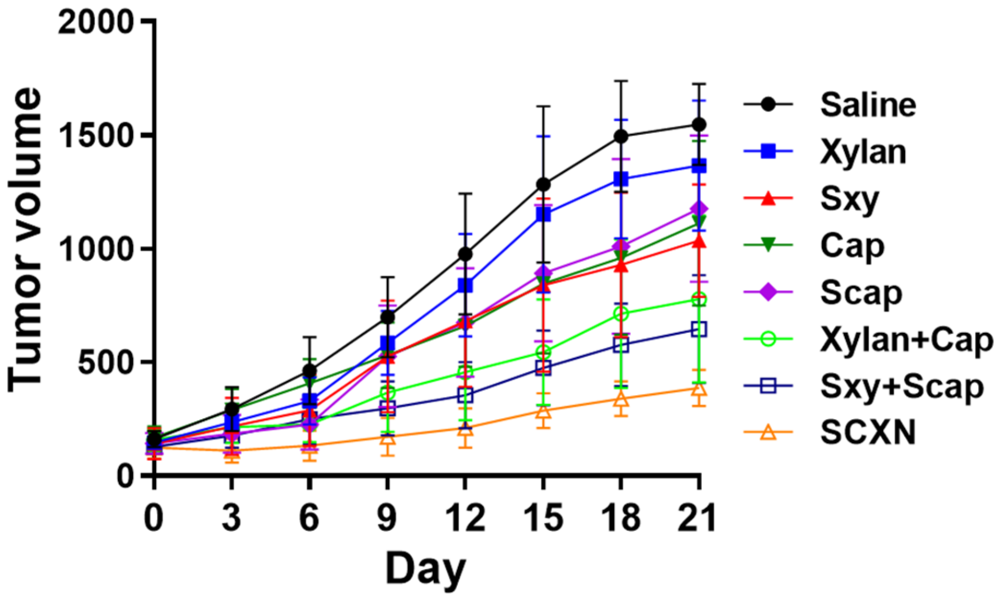

Supplementary Figure 6. The variation of average tumor volumes in the MC38 tumor-bearing mice during the therapy period. Data are shown as mean ± SD (n = 6 mice). Source data are provided as a Source Data file.

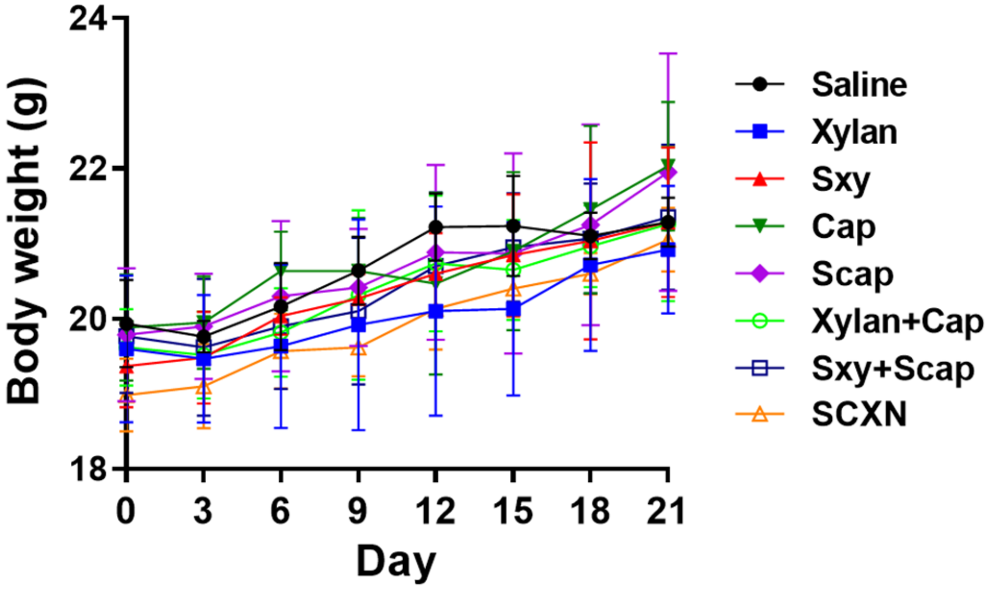

Supplementary Figure 7. The variation of average body weights of the MC38 tumor-bearing mice during the therapy period. Data are shown as mean ± SD (n = 6 mice). Source data are provided as a Source Data file.

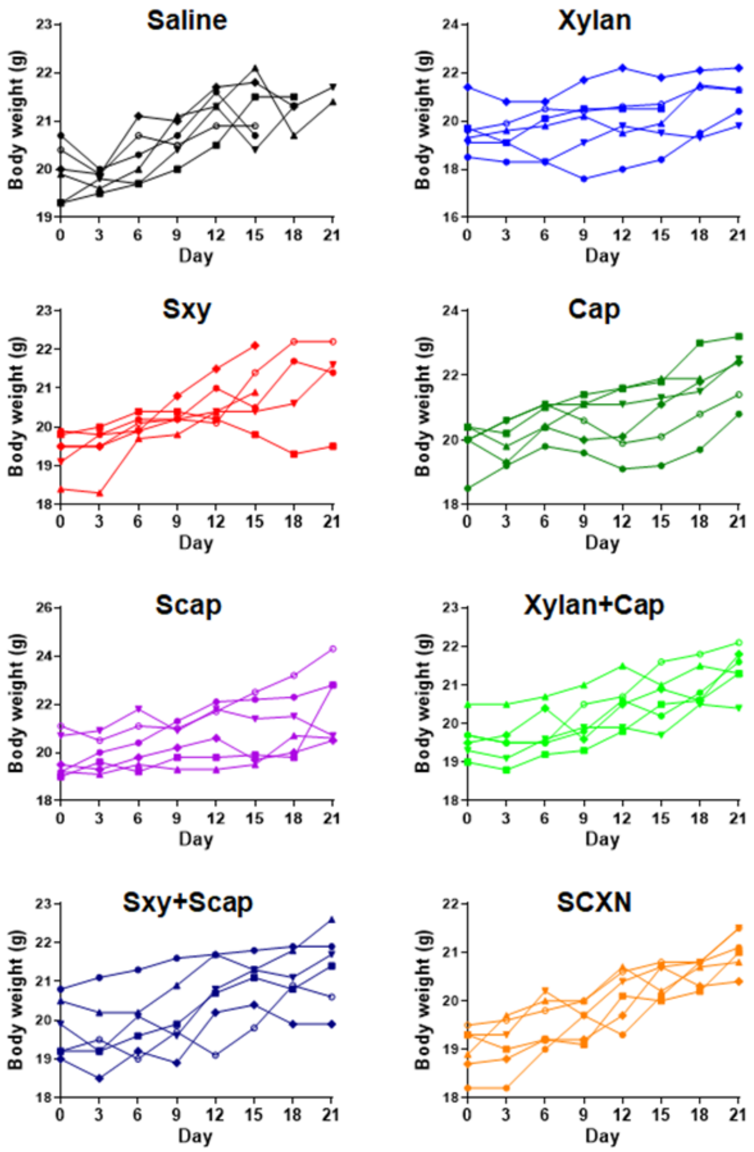

Supplementary Figure 8. The variation of body weight of each MC38 tumor-bearing mouse during the therapy period. n = 6 mice. Source data are provided as a Source Data file.

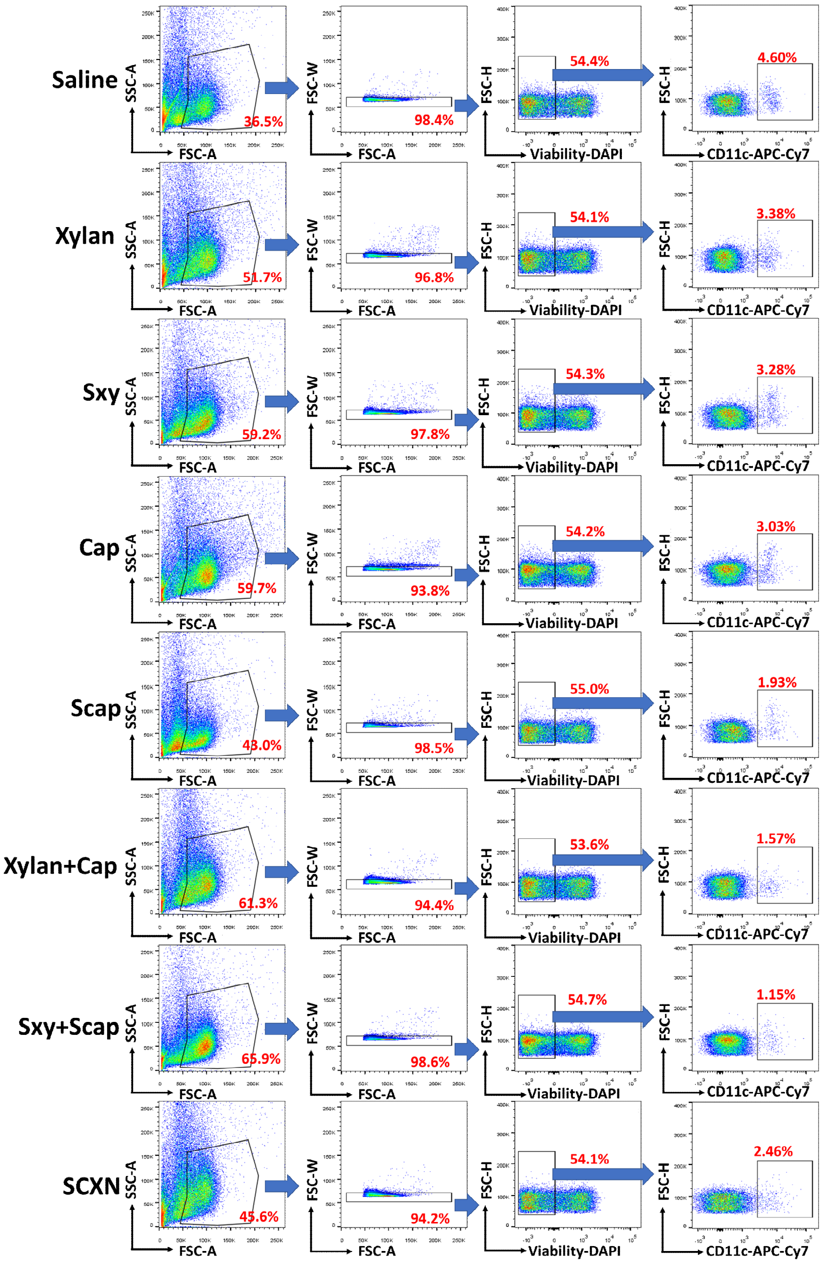

Supplementary Figure 9. The gating strategy for CD11c^{+} cells in the draining lymph nodes of the CT26 tumor-bearing mice.

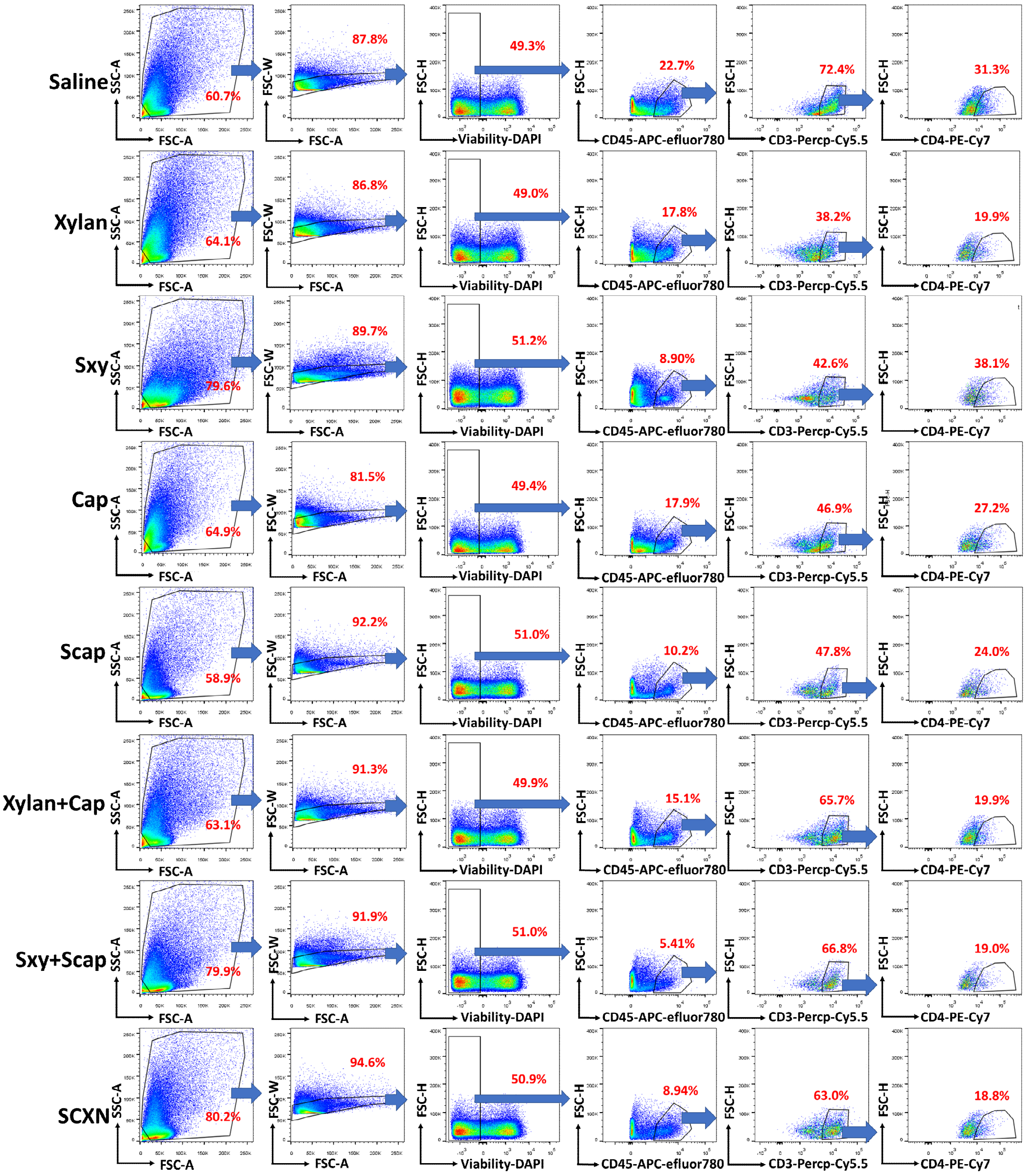

Supplementary Figure 10. The gating strategy for $CD3^+$ and $CD4^+$ cells in the tumors of the CT26 tumor-bearing mice.

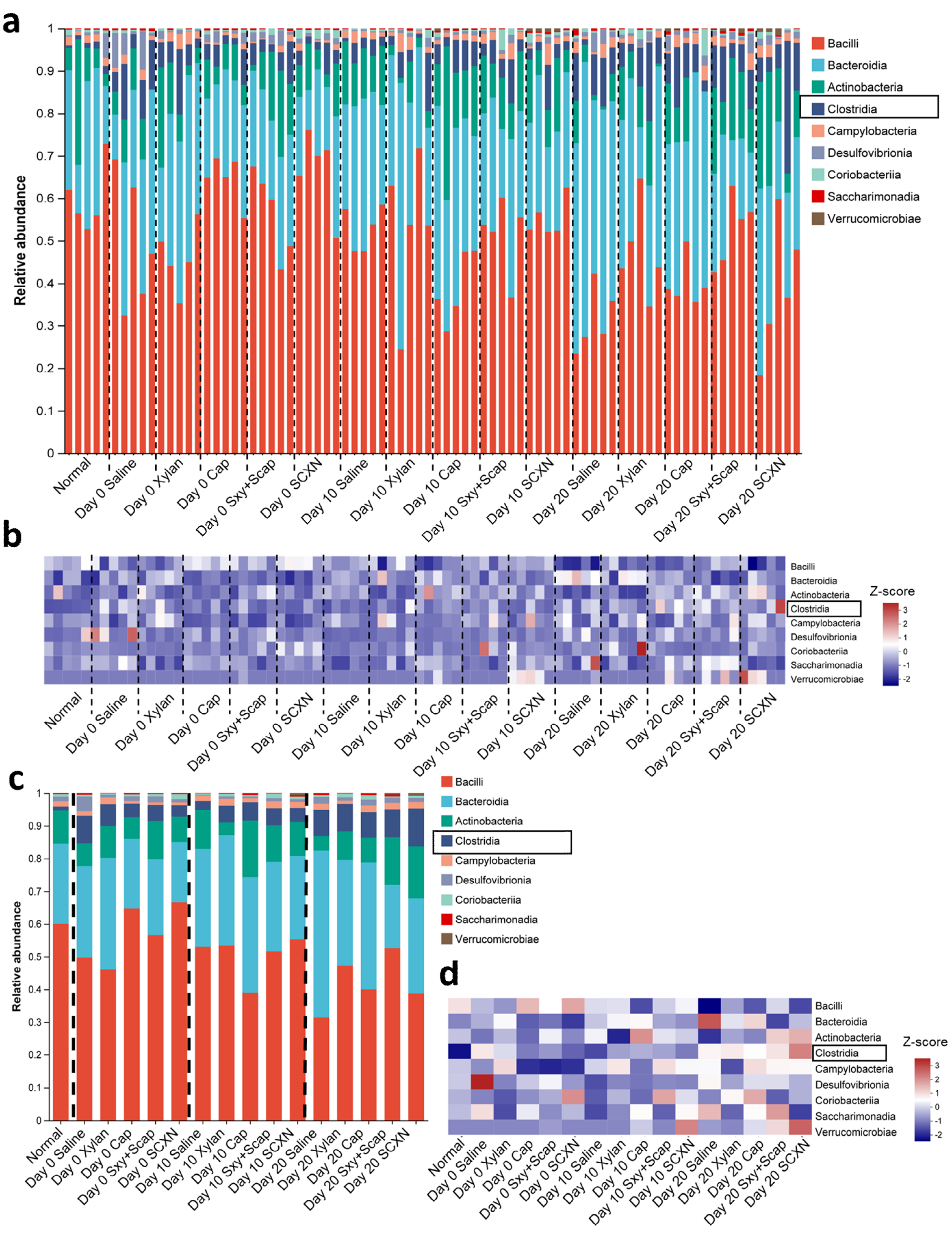

Supplementary Figure 11. The relative abundance at the class level of the intestine microbial community in CT-26 tumor-bearing mice receiving multi-dose of different formulations. **a, b,** Barplot (a) and heatmap (b) of samples from individual mice. **c, d,** Barplot (c) and heatmap (d) of samples from each group. n = 5 mice. Source data are provided as a Source Data file.

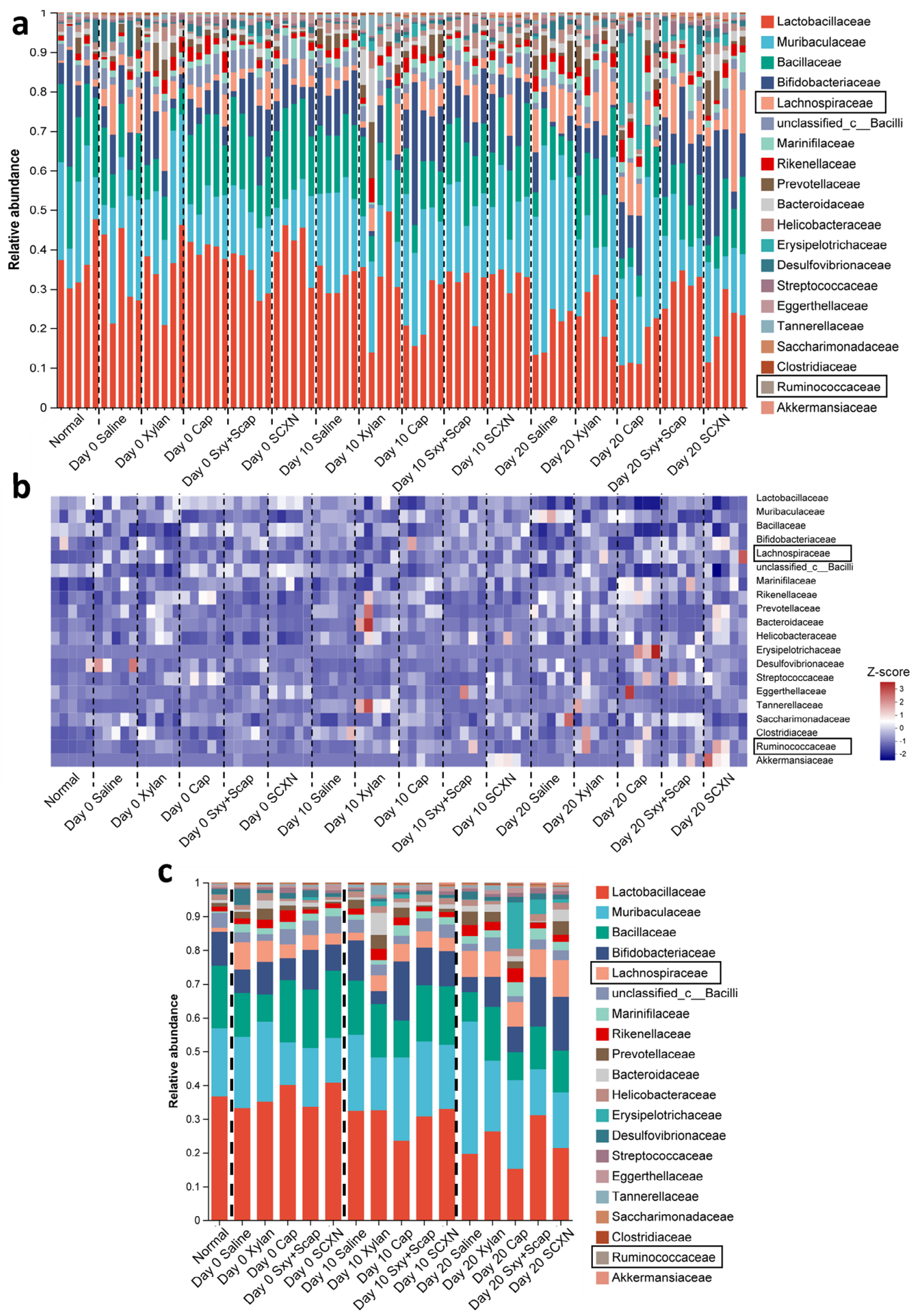

Supplementary Figure 12. The relative abundance at the family level of the intestine microbial community in CT-26 tumor-bearing mice receiving multi-dose of different formulations. **a, b,** Barplot (a) and heatmap (b) of samples from individual mice. **c,** Barplot of samples from each group. n = 5 mice. Source data are provided as a Source Data file.

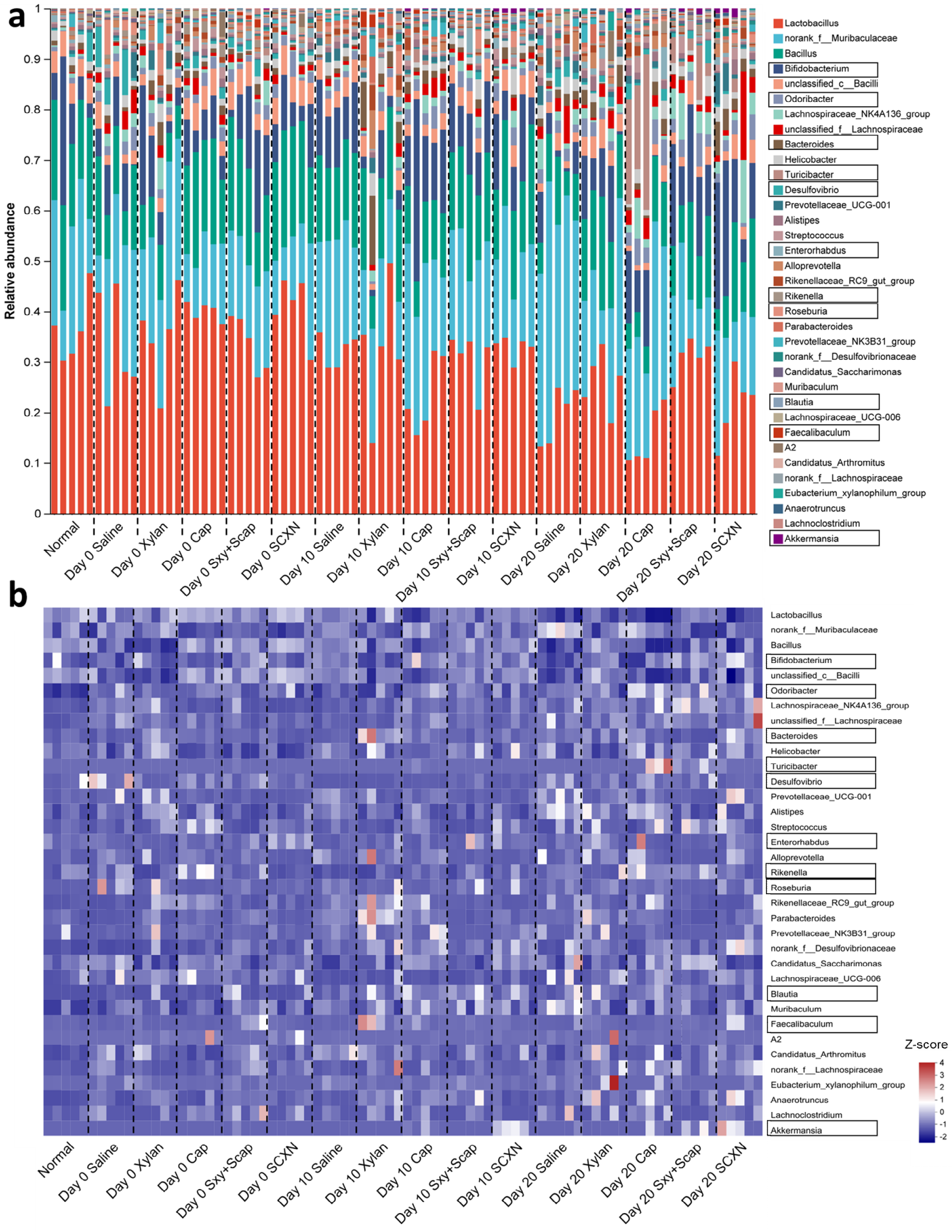

Supplementary Figure 13. The relative abundance at the genus level of the intestine microbial community in CT-26 tumor-bearing mice receiving multi-dose of different formulations. Heatmap (a) and barplot (b) of samples from individual mice. n = 5 mice. Source data are provided as a Source Data file.

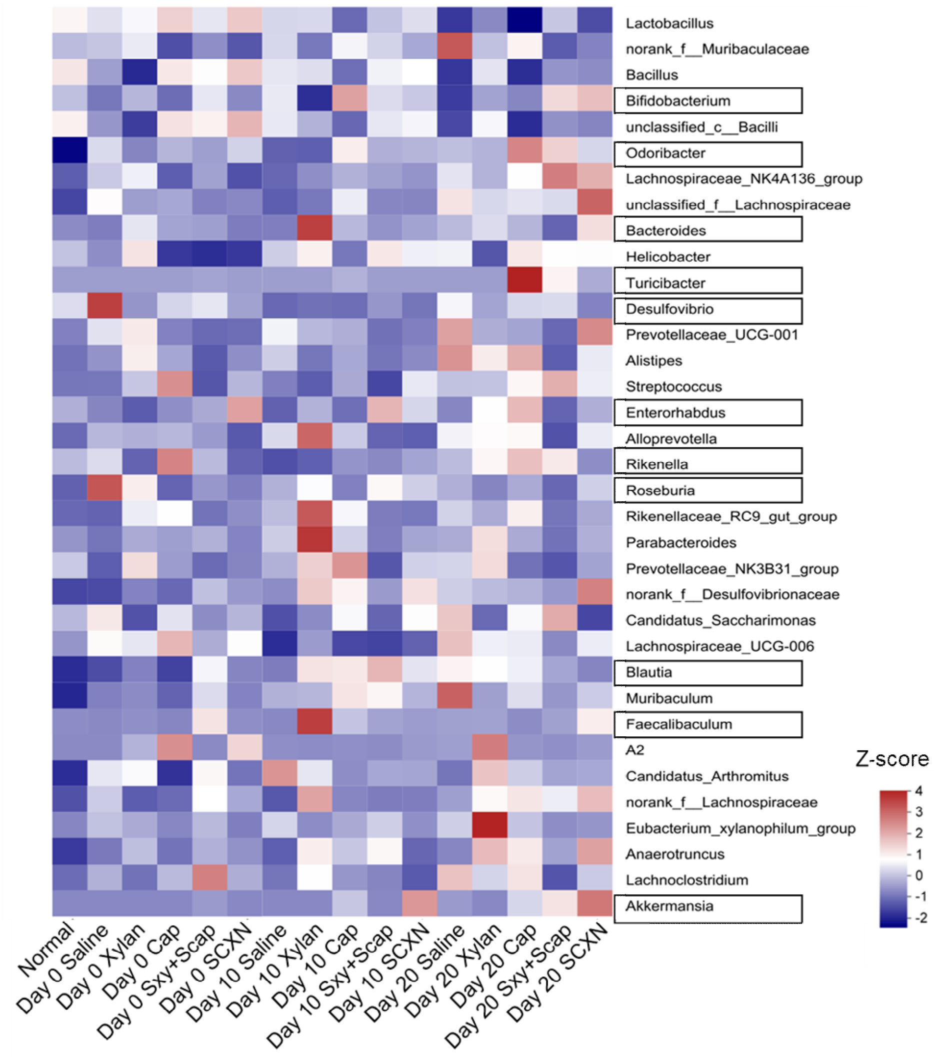

Supplementary Figure 14. Heatmap showing average relative abundance of the intestine microbial community at the genus level in CT-26 tumor-bearing mice receiving multi-dose of different formulations. n = 5 mice. Source data are provided as a Source Data file.

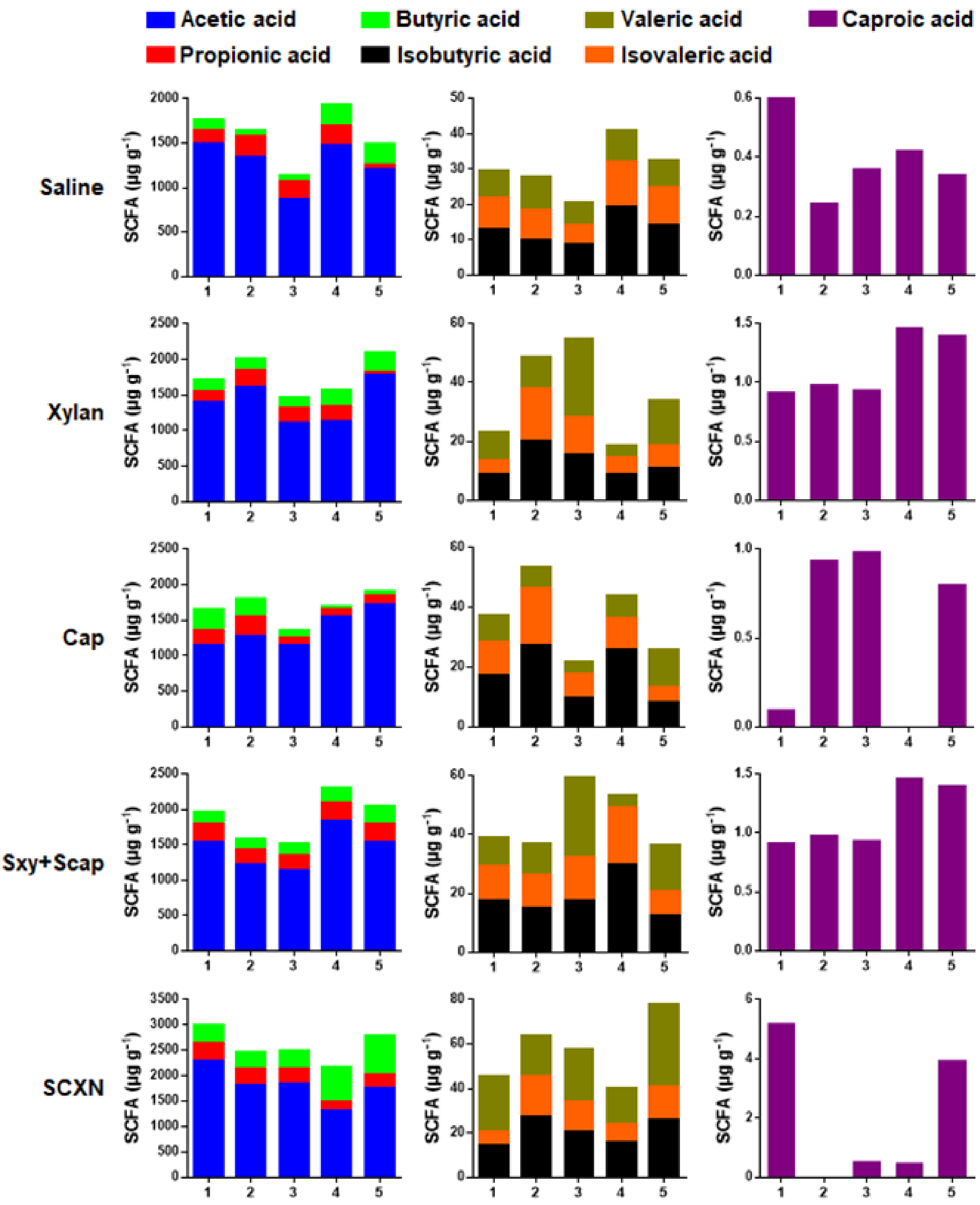

Supplementary Figure 15. SCFA levels in the faeces from individual CT-26 tumor-bearing mice receiving multi-dose of different formulations. n = 5 mice. Source data are provided as a Source Data file.

Supplementary Table 1. Pharmacokinetic parameters of Cap in mice after orally administrated with free Cap and SCXN at the dose of 100 mg kg^{-1} Cap. n = 3 mice.

Parameter	Cap	SCXN
C_{max} (μg mL^{-1})	2.90±0.25	4.19±0.36
$T_{1/2}$ (h)	2.48±0.09	6.78±1.08
$MRT_{(0-\infty)}$ (h)	3.59±0.25	7.94±0.33
$AUC_{(0-t)}$ (h·μg mL^{-1})	9.98±0.92	22.45±1.30
$AUC_{(0-\infty)}$ (h·μg mL^{-1})	10.44±0.90	24.11±1.06
Plasma clearance (mL h^{-1} kg^{-1})	64.10±5.82	27.68±1.53

Supplementary Table 2. Hematological parameters of the blood collected from mice treated with multi-dose of saline, xylan, Sxy, Cap, Scap, xylan+Cap, Sxy+Scap, or SCXN. n = 3 mice.

Group	RBC $[10^{12}\ L^{-1}]^{a}$	WBC $[10^{9}\ L^{-1}]^{b}$	PLT $[10^{9}\ L^{-1}]^{c}$	GRAN $[10^{9}\ L^{-1}]^{d}$	RDW $[\%]^{e}$	MCV $[fL]^{f}$
Saline	6.52±0.55	3.43±1.10	446.33±78.49	1.00±0.29	14.40±0.46	49.00±1.41
Xylan	6.84±0.32	3.93±2.21	409.00±58.39	0.87±0.09	15.77±2.28	46.43±1.19
Sxy	8.02±0.51	4.50±0.43	595.00±104.10	1.17±0.09	14.80±0.75	48.17±1.17
Cap	5.95±0.81	3.73±1.43	624.67±246.29	0.77±0.45	17.20±4.86	45.77±5.78
Scap	5.77±1.49	2.37±1.05	402.33±153.02	1.03±0.45	15.57±1.32	47.10±2.26
Xylan+Cap	7.42±0.27	2.30±0.53	429.67±57.57	0.8±0.1	21.03±9.59	44.60±6.18
Sxy+Scap	7.52±0.38	4.30±1.18	402.33±114.75	1.23±0.35	17.70±5.34	47.10±6.93
SCXN	8.05±0.25	4.20±0.69	571.33±108.15	1.20±0.40	14.40±0.51	49.73±0.71

a: red blood cells;

b: white blood cells;

c: platelets;

d: granulocytes

e: red cell distribution width;

f: mean corpuscular volume.
