# Supplementary material for: Combining gut microbiota modulation and chemotherapy by capecitabine-loaded prebiotic nanoparticle improves colorectal cancer therapy
Source: Nat Commun. 2023 Aug 7;14:4746. doi: 10.1038/s41467-023-40439-y (PMC10406894; doi:10.1038/s41467-023-40439-y)
Supplement: Supplementary file 3 — Reporting Summary [file 41467_2023_40439_MOESM3_ESM.pdf]

Corresponding author(s): Qi Yin, Yaping Li

Last updated by author(s): Jun 23, 2023

Reporting Summary

Nature Portfolio wishes to improve the reproducibility of the work that we publish. This form provides structure for consistency and transparency in reporting. For further information on Nature Portfolio policies, see our Editorial Policies and the Editorial Policy Checklist.

Statistics

For all statistical analyses, confirm that the following items are present in the figure legend, table legend, main text, or Methods section.

n/a	Confirmed	
☐	☒	The exact sample size (*n*) for each experimental group/condition, given as a discrete number and unit of measurement
☐	☒	A statement on whether measurements were taken from distinct samples or whether the same sample was measured repeatedly
☐	☒	The statistical test(s) used AND whether they are one- or two-sided<br>*Only common tests should be described solely by name; describe more complex techniques in the Methods section.*
☒	☐	A description of all covariates tested
☐	☒	A description of any assumptions or corrections, such as tests of normality and adjustment for multiple comparisons
☐	☒	A full description of the statistical parameters including central tendency (e.g. means) or other basic estimates (e.g. regression coefficient) AND variation (e.g. standard deviation) or associated estimates of uncertainty (e.g. confidence intervals)
☐	☒	For null hypothesis testing, the test statistic (e.g. *F*, *t*, *r*) with confidence intervals, effect sizes, degrees of freedom and *P* value noted<br>*Give P values as exact values whenever suitable.*
☒	☐	For Bayesian analysis, information on the choice of priors and Markov chain Monte Carlo settings
☒	☐	For hierarchical and complex designs, identification of the appropriate level for tests and full reporting of outcomes
☒	☐	Estimates of effect sizes (e.g. Cohen's *d*, Pearson's *r*), indicating how they were calculated

Our web collection on statistics for biologists contains articles on many of the points above.

Software and code

Policy information about availability of computer code

Data collection	Data were collected using the software of the instrument described in each experiment. FACSDiva 6.3.1 Software (flow cytometry), Spectrum Living Image 4.4.5 Software (live animal imaging), Zetasizer 7.11 Software (size and zeta potential), TEM Imaging & Analysis (transmission electron microscopy), MiSeq Illumina sequencing platform (16S rDNA sequencing).
Data analysis	All statistical analyses were performed on Graphpad Prism (version 8.3.0). The flow cytometry data were analyzed using FlowJo software package (version 10.6.2). Perkin Elmer Living image software was used to analyze bioluminescent and fluorescent images(version 4.5.5). The histological section images were analyzed using CaseViewer (version 2.0). 16S rDNA analysis of gut microbiota was performed using Qiime2 (version 2022.2) and monther software(version 1.30).

For manuscripts utilizing custom algorithms or software that are central to the research but not yet described in published literature, software must be made available to editors and reviewers. We strongly encourage code deposition in a community repository (e.g. GitHub). See the Nature Portfolio guidelines for submitting code & software for further information.

Data

Policy information about availability of data

All manuscripts must include a data availability statement. This statement should provide the following information, where applicable:

- Accession codes, unique identifiers, or web links for publicly available datasets
- A description of any restrictions on data availability
- For clinical datasets or third party data, please ensure that the statement adheres to our policy

All data generated or analysed during this study are included in this published article, Supplementary Information and Source data file. 16s rDNA sequencing data have been deposited in the NCBI Sequence Read Archive (https://www.ncbi.nlm.nih.gov/bioproject/PRJNA986432; accession number: PRJNA986432). Species taxonomic analysis refer to Silva 138/16s bacteria database (https://www.arb-silva.de/search/). H&E-stained tissue section images and flow cytometry data are available in the Science Data Bank (https://cstr.cn/31253.11.sciencedb.09258; accession code: 31253.11.sciencedb.09258). TEM images and LSCM images are available in the Science Data Bank (https://cstr.cn/31253.11.sciencedb.09096; accession code: 31253.11.sciencedb.09096).

Research involving human participants, their data, or biological material

Policy information about studies with human participants or human data. See also policy information about sex, gender (identity/presentation), and sexual orientation and race, ethnicity and racism.

Reporting on sex and gender	*Use the terms sex (biological attribute) and gender (shaped by social and cultural circumstances) carefully in order to avoid confusing both terms. Indicate if findings apply to only one sex or gender; describe whether sex and gender were considered in study design; whether sex and/or gender was determined based on self-reporting or assigned and methods used. Provide in the source data disaggregated sex and gender data, where this information has been collected, and if consent has been obtained for sharing of individual-level data; provide overall numbers in this Reporting Summary. Please state if this information has not been collected. Report sex- and gender-based analyses where performed, justify reasons for lack of sex- and gender-based analysis.*
Reporting on race, ethnicity, or other socially relevant groupings	*Please specify the socially constructed or socially relevant categorization variable(s) used in your manuscript and explain why they were used. Please note that such variables should not be used as proxies for other socially constructed/relevant variables (for example, race or ethnicity should not be used as a proxy for socioeconomic status). Provide clear definitions of the relevant terms used, how they were provided (by the participants/respondents, the researchers, or third parties), and the method(s) used to classify people into the different categories (e.g. self-report, census or administrative data, social media data, etc.) Please provide details about how you controlled for confounding variables in your analyses.*
Population characteristics	*Describe the covariate-relevant population characteristics of the human research participants (e.g. age, genotypic information, past and current diagnosis and treatment categories). If you filled out the behavioural & social sciences study design questions and have nothing to add here, write "See above."*
Recruitment	*Describe how participants were recruited. Outline any potential self-selection bias or other biases that may be present and how these are likely to impact results.*
Ethics oversight	*Identify the organization(s) that approved the study protocol.*

Note that full information on the approval of the study protocol must also be provided in the manuscript.

Field-specific reporting

Please select the one below that is the best fit for your research. If you are not sure, read the appropriate sections before making your selection.

☒ Life sciences ☐ Behavioural & social sciences ☐ Ecological, evolutionary & environmental sciences

For a reference copy of the document with all sections, see nature.com/documents/nr-reporting-summary-flat.pdf

Life sciences study design

All studies must disclose on these points even when the disclosure is negative.

Sample size	Sample size was chosen to ensure reproducibility of the experiments in accordance with the replacement, reduction and refinement principles of animal ethics regulation. Sample sizes employed in this study were referenced previously published studies (Nat Nanotech, 2021, 16, 1271–1280).No statistical method was used to predetermine sample size.
Data exclusions	No samples were excluded from the analyses.
Replication	All experimental findings were reliably reproduced. All in vitro and in vivo experiments were carried out with at least 3 biological replicates for each experimental group. Biological replicates are indicated within figure legends.

Randomization	All samples were randomly allocated into experimental groups.
Blinding	No blinding was used throughout experiments. The Investigators were not blinded to allocation during experiments and outcome assessment, and the data analyses were based on objectively measurable data.

Reporting for specific materials, systems and methods

We require information from authors about some types of materials, experimental systems and methods used in many studies. Here, indicate whether each material, system or method listed is relevant to your study. If you are not sure if a list item applies to your research, read the appropriate section before selecting a response.

Materials & experimental systems

n/a	Involved in the study
☐	☒ Antibodies
☐	☒ Eukaryotic cell lines
☒	☐ Palaeontology and archaeology
☐	☒ Animals and other organisms
☒	☐ Clinical data
☒	☐ Dual use research of concern
☒	☐ Plants

Methods

n/a	Involved in the study
☒	☐ ChIP-seq
☐	☒ Flow cytometry
☒	☐ MRI-based neuroimaging

Antibodies

Antibodies used

Anti-CD8 Rabbit Monoclonal Antibody (Servicebio, cat. no. GB13429, 1:800 dilution)
Anti-CD31 Mouse Monoclonal Antibody (Servicebio, cat. no. GB12063, 1:300 dilution)
Alexa488-conjugated Goat Anti-Rabbit IgG (Servicebio, cat. no. GB25303, 1:400 dilution)
CY3-conjugated Goat Anti-Mouse IgG (Servicebio, cat. no. GB21303, 1:300 dilution)
Anti-mouse CD16/CD32 antibodies (TONBO Bioscience, cat. no. 70-0161-U100, Clone: 2.4G2, 1:100 dilution).
APC-eFluor 780-Anti-mouse CD11c (eBioscience, cat. no. 12-0112-82, Clone: N418, 1:100 dilution)
PE-anti-mouse CD80 (eBioscience, cat. no. 12-0801-82, Clone: 16-10A1, 1:100 dilution)
APC-anti-mouse CD86 (eBioscience, cat. no. 17-0862-81, Clone: GL1, 1:100 dilution)
APC-eFluor 780-CD45 Rat Monoclonal Antibody (eBioscience, cat. no. 47-0451-80, Clone: 30-F11, 1:100 dilution)
PerCP-Cy5.5-anti-mouse CD3 (TONBO Bioscience, cat. no. 65-0031-U100, Clone: 145-2C11, 1:100 dilution)
PE-Cy7-anti-mouse CD4 (BioLegend, cat. 100527, Clone: RM4-5, 1:100 dilution)
APC-anti-mouse CD8a (BioLegend, cat. no. 100711, Clone: 53-6.7, 1:100 dilution)
PE-anti-mouse Foxp3 (TONBO Bioscience, cat. no. 50-5773-U025, Clone: 3G3, 1:100 dilution)

Validation

anti-CD8 Rabbit Monoclonal Antibody
https://shoposs.servicebio.cn/d/file/p/20200611/4a422e02568648e76b22abb6929e9e13.pdf

anti-CD31 Mouse Monoclonal Antibody
https://shoposs.servicebio.cn/2023/05/24/1684914947824vsNZzs.pdf

Alexa488-conjugated Goat Anti-Rabbit IgG
https://shoposs.servicebio.cn/d/file/p/20200508/2d79ecef85445afaad1a856b42a6db65.pdf

CY3-conjugated Goat Anti-Mouse IgG
https://shoposs.servicebio.cn/d/file/p/20200508/2d79ecef85445afaad1a856b42a6db65.pdf

Anti-mouse CD16/CD32 antibodies
https://cytekbio.com/products/purified-anti-mouse-cd16-cd32-2-4g2-fc-block

APC-eFluor 780-Anti-mouse CD11c
https://www.thermofisher.cn/cn/zh/antibody/product/CD11b-Antibody-clone-M1-70-Monoclonal/12-0112-82

PE-anti-mouse CD80
https://www.thermofisher.cn/cn/zh/antibody/product/CD80-B7-1-Antibody-clone-16-10A1-Monoclonal/12-0801-82

APC-anti-mouse CD86
https://www.thermofisher.cn/cn/zh/antibody/product/CD86-B7-2-Antibody-clone-GL1-Monoclonal/17-0862-81

APC-eFluor 780-CD45 Rat Monoclonal Antibody
https://www.thermofisher.cn/cn/zh/antibody/product/CD45-Antibody-clone-30-F11-Monoclonal/47-0451-80

PerCP-Cy5.5-anti-mouse CD3
https://cytekbio.com/products/percp-cyanine5-5-anti-mouse-cd3e-145-2c11

PE-Cy7 anti-mouse CD4
https://www.biolegend.com/en-us/products/pe-cyanine7-anti-mouse-cd4-antibody-193

APC-anti-mouse CD8a
https://www.biolegend.com/en-us/products/apc-anti-mouse-cd8a-antibody-150),

PE-anti-mouse Foxp3
https://cytekbio.com/products/pe-anti-mouse-foxp3-3g3

Eukaryotic cell lines

Policy information about cell lines and Sex and Gender in Research

Cell line source(s)	CT26 murine colon carcinoma cells and CT26-luc cells were kindly provided by Prof. Yongzhuo Huang's lab in Shanghai Institute of Materia Medica, CAS. MC38 murine colon carcinoma cells (cat. no. FH0125) were obtained from Fuheng Biology Co. Ltd (Shanghai, China).
Authentication	These cell lines were authenticated by the supplier using STR analysis.
Mycoplasma contamination	No mycoplasma contamination was found.
Commonly misidentified lines (See ICLAC register)	These all cell lines that we used were not listed in commonly misidentified lines in ICLAC register.

Animals and other research organisms

Policy information about studies involving animals; ARRIVE guidelines recommended for reporting animal research, and Sex and Gender in Research

Laboratory animals	The female Balb/c mice aged 4-6 weeks (18-22 g) and female C57BL/6 mice aged 4-6 weeks (18-22 g) were obtained from Beijing Charles River Laboratory Animal Technology Co., Ltd (Beijing, China) and raised in the animal care facility in the SPF grade environment with sterilized food pellets and distilled water under a 12 h light/dark cycle. The normal chow included ≥ 18% protein, ≥ 4% fat, ≤ 5% crude fibre, ≤ 8% ash, 1.0-1.8% Calcium and less than 10% moisture (Keao Xieli Feed Co.,Ltd., cat. no. 1016706714625204224). The temperature range for the housing room was ~24 °C and the humidity range was ~50%.
Wild animals	The study did not involve wild animals.
Reporting on sex	We did not consider the influence of sex in the study design. Female mice were used for all the animal assay as reported in the literature studies.
Field-collected samples	The study did not involve samples collected from the field.
Ethics oversight	All animal procedures were performed with ethical compliance and approved by the Institutional Animal Care and Use Committee of the Shanghai Institute of Materia Medica, Chinese Academy of Sciences.

Note that full information on the approval of the study protocol must also be provided in the manuscript.

Flow Cytometry

Plots

Confirm that:

- [x] The axis labels state the marker and fluorochrome used (e.g. CD4-FITC).
- [x] The axis scales are clearly visible. Include numbers along axes only for bottom left plot of group (a 'group' is an analysis of identical markers).
- [x] All plots are contour plots with outliers or pseudocolor plots.
- [x] A numerical value for number of cells or percentage (with statistics) is provided.

Methodology

Sample preparation	For DC maturation level evaluation, the lymph nodes were grinded in PBS (1 mL), filtered with nylon nets, and then centrifuged at 500 g for 15 min. After the supernatant was removed, 300 µL of PBS was added to resuspend cells. The cells were washed with PBS, stained with viability dye, and blocked with Anti-mouse CD16/CD32 antibodies. The cells were then stained with the designated antibodies.<br>To investigate the tumor-infiltration of T lymphocytes, the tumor tissues were crushed, and treated with 2 mL of the mixture solution of 1 mg mL-1 collagenase IV, 1 mg mL-1 hyaluronidase, and 0.2 mg mL-1 DNase I for 2 h at 37℃. After removing the

	tumor debris with nylon nets, the filtrate was centrifuged at 500 g for 5 min, then the supernatant was removed. The cells were washed with PBS, stained with viability dye, and blocked with Anti-mouse CD16/CD32 antibodies. The cells were then stained with the designated antibodies.
Instrument	BD Fortessa flow cytometer (USA)
Software	BD FACSDiva software (version 6.3.1) were used for data collection, and Flowjo software package (version 10.6.2) was used for data analysis.
Cell population abundance	Cell populations of interest were collected as much as possible in the intratumoural immunology assay. No post-sort fractions were collected. Flow cytometry was used for quantification only.
Gating strategy	Cells were gated first by morphology to exclude cell debris, doublets were then gated out by FSC-A/FSC-W, followed by exclusion of dead cells by gating on dye negative cells.

☒ Tick this box to confirm that a figure exemplifying the gating strategy is provided in the Supplementary Information.
